# Antimicrobial Properties of Microparticles Based on Carmellose Cross-Linked by Cu^2+^ Ions

**DOI:** 10.1155/2015/790720

**Published:** 2015-05-19

**Authors:** Martina Kejdušová, Jakub Vysloužil, Kateřina Kubová, Vladimír Celer, Magdaléna Krásna, Alena Pechová, Věra Vyskočilová, Vratislav Košťál

**Affiliations:** ^1^Department of Pharmaceutics, Faculty of Pharmacy, University of Veterinary and Pharmaceutical Sciences Brno, Palackého Třída 1/3, 612 42 Brno, Czech Republic; ^2^Department of Infectious Diseases and Microbiology, Faculty of Veterinary Medicine, University of Veterinary and Pharmaceutical Sciences Brno, Palackého 1/3, 612 42 Brno, Czech Republic; ^3^Department of Biochemistry and Biophysics, Faculty of Veterinary Hygiene and Ecology, University of Veterinary and Pharmaceutical Sciences Brno, Palackého 1/3, 612 42 Brno, Czech Republic; ^4^Tescan, Libušina Třída 863/21, 623 00 Brno-Kohoutovice, Czech Republic

## Abstract

Carmellose (CMC) is frequently used due to its high biocompatibility, biodegradability, and low immunogenicity for development of site-specific or controlled release drug delivery systems. In this experimental work, CMC dispersions in two different concentrations (1% and 2%) cross-linked by copper (II) ions (0.5, 1, 1.5, or 2.0 M CuCl_2_) were used to prepare microspheres with antimicrobial activity against *Escherichia coli* and *Candida albicans*, both frequently occurring pathogens which cause vaginal infections. The microparticles were prepared by an ionotropic gelation technique which offers the unique possibility to entrap divalent copper ions in a CMC structure and thus ensure their antibacterial activity. Prepared CMC microspheres exhibited sufficient sphericity. Both equivalent diameter and copper content were influenced by CMC concentration, and the molarity of copper (II) solution affected only the copper content results. Selected samples exhibited stable but pH-responsive behaviour in environments which corresponded with natural (pH 4.5) and inflamed (pH 6.0) vaginal conditions. All the tested samples exhibited proven substantial antimicrobial activity against both Gram-negative bacteria *Escherichia coli* and yeast *Candida albicans*. Unexpectedly, a crucial parameter for microsphere antimicrobial activity was not found in the copper content but in the swelling capacity of the microparticles and in the degree of CMC surface shrinking.

## 1. Introduction

Carmellose (CMC) is a water-soluble anionic polysaccharide and semisynthetic derivative of cellulose [1] which has no harmful effects on human health. Its chains are linear *β*(1 → 4)-linked glucopyranose units. In addition, it contains a hydrophobic polysaccharide backbone and many hydrophilic carboxyl groups and, as a result, exhibits amphiphilic characteristics. CMC is used in a number of applications throughout the food, cosmetic, textile, paper, and ceramic industries as a viscosity modifier, thickener, emulsion stabilizer, and water retention and adhesive agent [2]. It also has tremendous potential for use in pharmaceutical products including site-specific or controlled release drug delivery carrier matrices thanks to its high biocompatibility, biodegradability [3], and low immunogenicity [4]. In spite of the numerous positive properties of CMC, it does not have any antimicrobial properties as it lacks antimicrobial functional groups [5]. To bestow CMC with antimicrobial properties, different possibilities have been proposed, such as (i) adding different antimicrobial agents, for example, potassium sorbate [6] or silver nitrate [7], (ii) grafting CMC with different antimicrobial substances, for example, guanidine hydrochloride [8], (iii) combining CMC with other polymers that exhibit antimicrobial properties, for example, carboxymethyl chitosan [9, 10], or (iv) preparing nanoparticles of certain metals, for example, with silver [7] or copper [11], and incorporating them into the CMC structure.

Copper is one of the most abundant trace elements found in the human body. It is an essential nutrient involved in catalyzing biochemical reactions. However, excessive copper levels can be toxic, mainly because they cause oxidative damage to the body. Copper can change its redox status by accepting and donating electrons, shifting between cuprous (Cu^+^) and cupric (Cu^2+^) forms, and therefore participate in reactions that generate superoxide radicals and hydrogen peroxide, which are major reactive oxygen species in the body. That is why a systemic usage of copper is limited in the human body with exception of rheumatoid arthritis treatment [12–14]. New options for copper utilization can be found in its local effect via vaginal application of dosage forms based on copper ions for their strong antibacterial and spermicidal and/or spermiostatic effect. The vagina produces fluid at a rate of 3-4 g/4 h [15] which can reduce the potent local toxic effect of the copper compounds. Mucus efficiently traps foreign particles and particulates by both steric and adhesive mechanisms, facilitating rapid clearance [16]. Daunter used copper ethylenediaminetetraacetic acid/L-ascorbic acid as a fertilization-preventing agent that can be delivered via nonbiodegradable, polyurethane, or polyvinyl acetate vaginal discs [17].

An aqueous CMC solution can undergo a sol-gel transformation in the presence of cross-linking cations and thus allow for the creation of solid gel microparticles. This method is known as an ionotropic gelation technique and is based on the impact of physical (electrostatic) forces and polyelectrolyte complexation with the presence of polyvalent ions [18]. In general, divalent cations (e.g., Ba^2+^, Sr^2+^, Cd^2+^, Co^2+^, Ca^2+^, Mn^2+^, Ni^2+^, Pb^2+^, and Zn^2+^) are suitable cross-linking agents for this method [19]. Thus, ionotropic gelation offers a unique possibility to incorporate divalent copper ions into the CMC structure and form microspheres with potential antimicrobial properties.

The aim of the presented research was to prepare Cu^2+^ cross-linked CMC microspheres using external ionic gelation and assess their antimicrobial activity against Gram-negative* Escherichia coli* and* Candida albicans* yeast, both frequently occurring pathogens responsible for vaginal infections.

## 2. Materials and Methods

### 2.1. Materials

Blanose-carmellose (CMC) with a medium viscosity grade (1500–3100 mPa·s for 2% in water) and a DS of 1.2 was used as the polymer carrier (Ashland, Covington, USA), copper (II) chloride was used as a cross-linking agent (Sigma Aldrich, St. Louis, USA), and HNO_3_ (65% v/v) and H_2_O_2_ (30% v/v) used for the determination of copper content were purchased from Analytika (Prague, Czech Republic). Calibration solutions were prepared using a dilution of 1000 mg/L stock copper reference solvent (Analytika, Prague, Czech Republic). Deionised water with a resistivity of 18 MΩ was used for all necessary dilution.

### 2.2. Methods

#### 2.2.1. Microparticles Preparation

Copper cross-linked CMC microparticles were prepared by a method of external ionic gelation. CMC dispersions (1% and 2%) were prepared by dispersing 1 g and 2 g of CMC in purified water, respectively. The dispersions were heated to 80°C to increase the rate of swelling. They were then homogenized using an Ultra-Turrax (T25 basic, IKA-Werke, Staufen, Germany) at 13,000 rpm for 5 min. The volume of each dispersion was ultimately adjusted to 100 mL with purified water. The resulting dispersions were then extruded through a needle with an internal diameter of 0.4 mm at a dropping rate of 2.0 mL/min into 50 mL of 0.5, 1.0, 1.5, or 2.0 M  CuCl_2_ aqueous solution, respectively. The distance between the edge of the needle and the surface of the solution was adjusted to 5.0 cm. Microparticle formation was instantaneous and the particles were left in the cross-linking solution for 1 hour to harden while being gently mixed. The resulting beads were subsequently washed three times with purified water and dried at 25°C in a cabinet drier (HORO –048B, Dr. Hofmann GmbH, Ostfildern, Germany) for 24 hrs before testing. Prepared samples were named in accordance with the type and values of the altered variables (CMC concentration and molarity of the cross-linking medium). Samples characteristics are shown in Table 1.

#### 2.2.2. Viscosity Measurement

To investigate the influence of the viscosity on the characteristics of the prepared microparticles, rheological measurements of CMC dispersions were performed. Dispersions (1.0 and 2.0 wt%) were prepared and then homogenized with Ultra-Turrax (T25 BASIC, IKA-Werke GmbH & Co. KG, Staufen, Germany) at 16,000 rpm. Dynamic viscosity was measured by a Brookfield DV-II+Pro rotary viscometer (Brookfield Engineering, Middleboro, USA) and Rheocalc software (Brookfield Engineering, Middleboro, USA) at 37°C and 200 rpm. A small sample adapter was employed for this step. Each sample was measured three times and the results were expressed as mean values with standard deviations (SD).

#### 2.2.3. Scanning Electron Microscopy

In order to observe microparticle morphology and surface topography, scanning electron microscopy (SEM) was employed. The samples were mounted directly onto the SEM sample holder using double-sided sticking tape and then coated with a 10 nm thick layer of Au. Images were taken using the MIRA3 scanning electron microscope (Tescan, Brno, Czech Republic) at an accelerating voltage of 5.0 kV.

#### 2.2.4. Optical Microscope Analysis

Particle size of the copper microparticles was measured using a NIKON SMZ 1500 stereoscopic microscope (Nikon, Tokyo, Japan) equipped with a 72AUC02 USB camera (The Imaging Source, Bremen, Germany). Microparticles were visualized under ×15 magnification. The obtained images of 200 randomly chosen microparticles were stored and subsequently processed using the NIS-Elements AR 4.0 computer software (Nikon, Tokyo, Japan).

Equivalent diameter (ED) and sphericity factor (SF) were calculated from the measured values and expressed as arithmetic mean ± standard deviation.

#### 2.2.5. Copper Content in Microparticles

The copper content in the prepared microparticles was determined by atomic absorption. To digest the microparticles, 6 mL of concentrated nitric acid (65% v/v) and 2 mL of hydrogen peroxide (30% v/v) were added to 10 mg of every sample and placed in a TFM digestion vessel. The vessels were closed and placed into the segment and the content was mineralized using an Ethos SEL Microwave Labstation (Milestone, Italy) at 220°C for 35 min, applying a maximal power of 1000 W. The microwave programme was started by steadily increasing the temperature over 15 min, followed by holding the temperature for an additional 20 min. After cooling, each resulting solution was transferred to 50 mL glass flasks and filled to the mark with deionized water. Samples were diluted to 1 : 19 with deionized water prior to further analysis. Copper content was measured using air-acetylene flame atomization in a contrAA 700 atomic absorption spectrometer (Analytik Jena, Germany). All samples were measured in triplicate and the obtained values were processed by Aspect CS software, version 2.1.

#### 2.2.6. Swelling Capacity

To determine swelling capacity, a previously reported method was improved [20]. The test was performed in a medium that properly simulates vaginal conditions. 100 mg of each sample was put into fine mesh baskets and immersed in a pH 4.5 medium (natural vaginal environment—6.80 g of potassium dihydrogen phosphate R in 1000.0 mL of water R) and a pH 6.0 medium (infected vaginal environment—6.8 g of sodium dihydrogen phosphate R in 1000.0 mL of water R, pH adjustment with strong sodium hydroxide solution R). The baskets were pulled out at time intervals of 5, 10, 15, 30, and 45 min and 1, 2, 3, 4, 5, and 6 hours after the first immersion, properly dried, and weighed. Swelling capacity was calculated using the following equation [21]:

(1) $$\begin{array}{c} S_{\text{SW}}=\left(\frac{W_{t}-W_{0}}{W_{0}}\right)\times 100\left(\%\right). \end{array}$$

*S*_SW_ is swelling capacity expressed as a percentage of weight addition, *W*_*t*_ is the weight of a sample at the relevant time interval, and *W*_0_ represents the initial weight of the sample. For each batch, the measurement was performed three times and results were expressed as mean values with standard deviations.

#### 2.2.7. Antimicrobial Activity: Bacterial Strains and MIC Determination

*E. coli* (CCM 4517) was maintained in a blood agar. To determine minimal inhibition concentration (MIC), an overnight culture of* E. coli* cells was suspended in a fresh LB (Luria broth) medium and grown to OD_600_ 0.5 at 37°C at a shaking speed of 250 rpm.

*Candida albicans* (CCM 8186) was maintained in an YNB (Yeast Nitrogen Base with ammonium sulfate) medium at 37°C. For MIC determination, an overnight culture was suspended in a fresh YNB medium and grown to OD_600_ 0.5 at 37°C at a shaking speed of 250 rpm.

A suitable amount of CMC microparticles was suspended in the growth media (LB medium for* E. coli*, YNB for* C. albicans*) to prepare a 10% suspension and incubated for 60 min at room temperature to release the copper into solution. Undissolved particles were then separated by centrifugation and discarded and atomic absorption was used to determine copper concentration. Twofold dilutions of copper suspension were prepared in appropriate mediums in a microtiter plate (100 *μ*L/well in triplicate). Then, 10e6 cells (*E. coli* or* C. albicans*) were added to each well of the microtiter plate and incubated at 37°C. To monitor cell growth, optical density was measured spectrophotometrically (OD_600_) after 20 hrs of incubation. The mean of the three wells was calculated to evaluate antimicrobial activity. Wells containing the* E. coli* or* C. albicans* without copper inhibition were included in triplicate in all tested plates as controls. The MIC value was expressed as the concentration of copper in a well showing at least a fourfold reduction of OD_600_ absorbance compared to each subsequent well.

## 3. Results and Discussion

**Figure 1:**
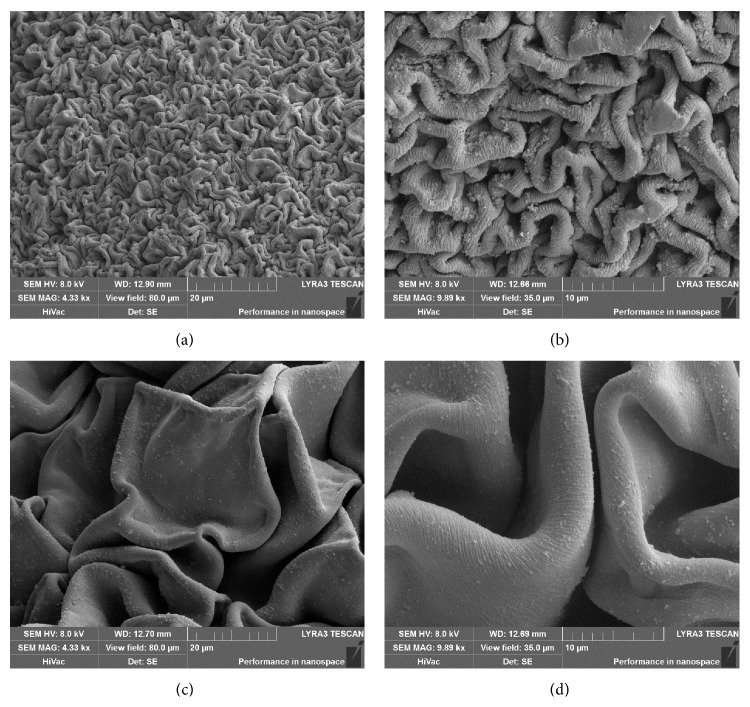
SEM photographs of surface topography of CMC microparticles: (a) MP-1_0.5 (magnification 4330x), (b) surface detail of MP-1_0.5 (magnification 9890x), (c) MP-2_0.5 (magnification 4330x), and (d) surface detail of MP-2_0.5 (magnification 9890x).

The copper cross-linked carmellose microparticles were evaluated for particle size, sphericity factor, and copper content (Table 2). The equivalent diameter of the prepared particles ranged from 738.1 ± 30.3 to 1078 ± 12.4 *μ*m. It seems that particle size did not depend on the concentration of the Cu^2+^ hardening solution. However, it was observed that it did increase with increasing CMC concentration which can be attributed to the increased viscosity [22, 23]. Viscosity of the 1% CMC dispersion was found to be 162 ± 0.21 mPa·s and that of the 2% CMC dispersion 766.67 ± 0.47 mPa·s. A polymer dispersion with higher viscosity is more difficult to form into smaller droplets [24]; thus these dispersions yielded larger particles. Another influence on the particle size of microspheres could be seen in the degree of CMC chain shrinkage. Generally, particle size decreases inversely with the degree of shrinkage. It has been well documented that the degree of shrinkage is typically higher for beads prepared from a dispersion with lower polymer concentration [25, 26]. A comparison of the microparticle surfaces (MP-1_0.5; ED = 775.7 *μ*m versus MP-2_0.5; ED = 1016.9) in Figure 1 confirms these findings. From these SEM photographs, it is evident that the degree of CMC shrinkage was significantly higher for the smaller 1% CMC microspheres.

Prepared CMC microspheres exhibited sufficient sphericity, ranging from 0.850 ± 0.067 to 0.934 ± 0.038. Previous studies indicate that particles with this parameter value above 0.8 are considered to have good sphericity [27]. It can be observed from the results that the sphericity was not clearly influenced by increasing the concentration of the hardening solution, but it was noticed that hardening solutions with lower molarity of CuCl_2_ (0.5 and 1.0%) yielded microspheres with higher sphericity values. On the other hand, particle sphericity was lower in samples prepared with a higher polymer concentration, with the exception of samples MP-1_2.0 and MP-2_2.0, which were prepared with 2 M of CuCl_2_ hardening solution. Previously reported results confirmed that increasing the concentration of CMC solutions linearly increases the solution viscosity [28] and sphericity of particles. Our results generally coincide with findings that overly viscous polymer solutions (2% in our case) form less spherical, tail-shaped particles [29] and particles with a rough surface [30].

Table 2 also shows the results of atomic absorption analysis for copper content. Copper content in the prepared microparticles ranged from 101.3 ± 0.65 to 200.3 ± 1.03 g/kg. It was observed that the content significantly increased with increases in hardening solution concentration as well as polymer concentration.

**Figure 2:**
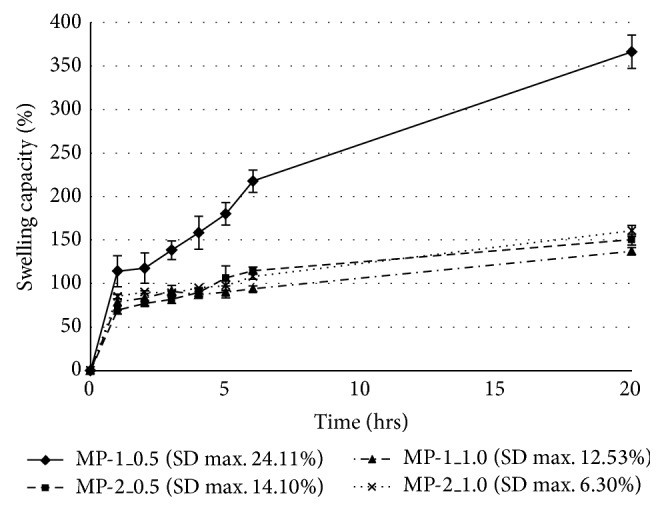
Degree of swelling in Cu^2+^ cross-linked microparticles at pH 4.5.

**Figure 3:**
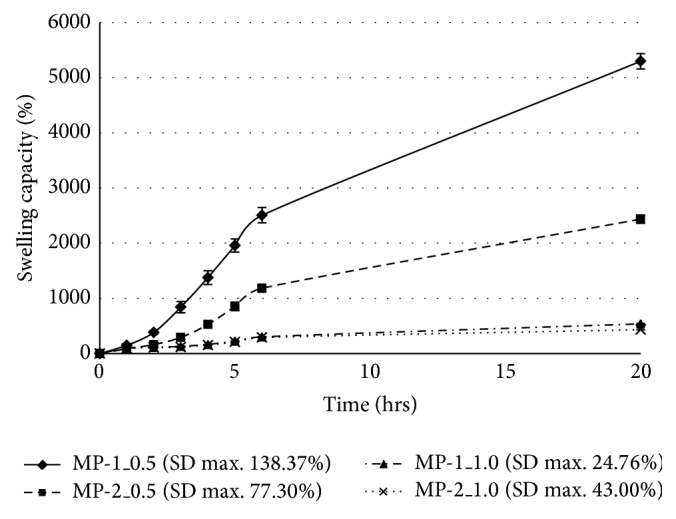
Degree of swelling in Cu^2+^ cross-linked microparticles at pH 6.0.

**Figure 4:**
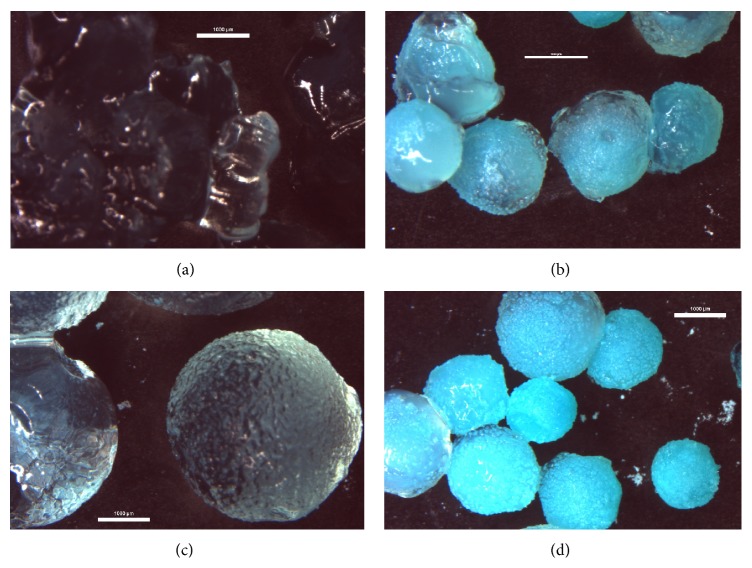
Optical microscope images of CMC particles after 20-hour swelling capacity test in pH 6.0 buffer; bars correspond to 1000 *μ*m: (a) MP-1_0.5, (b) MP-1_1.0, (c) MP-2_0.5, and (d) MP-2_1.0.

The samples of microspheres with the highest sphericity (MP-1_0.5, MP-1_1.0, MP-2_0.5, and MP-2_1.0), which is one the most important criteria for the preparation of particle systems, were then investigated for swelling capacity in phosphate buffers with pH 4.5 [31] and pH 6.0 [32], respectively, to simulate natural and inflamed vaginal conditions and to predict their behaviour on vaginal mucosa for a 20-hour time interval (20-hour interval represents an estimated time of therapy). The obtained results can be seen in Figure 2 for pH 4.5 and in Figure 3 for pH 6.0.  Figure 4 shows images of microparticles after a 20-hour swelling capacity test in a pH 6.0 buffer. Generally, it was observed that the swelling capacity of all samples gradually increased during the test. Selected samples exhibited pH-responsive behaviour which differed based on the tested environments; swelling was substantially higher at pH 6.0 [33]. It is obvious from Figures 2 and 3 that sample MP-1_0.5, prepared with a less-concentrated hardening solution (0.5 M) and a lower CMC concentration (1%), had the highest swelling capacity at both pH values (366.2% at pH 4.5 after 20 hrs, 5296.3% at pH 6.0 after 20 hrs). These values of formulation variables led to the creation of favourable conditions for water uptake into the microsphere matrix, resulting in the creation of an amorphous gel structure at pH 6.0 (see Figure 4(a)). Also, the enormous loss of the blue colour in comparison with sample MP-2_1.0 (Figure 4(d)) can be noticed, probably related to high water uptake and higher release of the copper ions which are responsible for the blue colour. This is uniquely in accordance with previously published data saying that increased polymer or hardening solution concentrations can significantly reduce water uptake due to the increased density of the polymer network [34]. At pH 6.0, sample MP-2_0.5 followed with swelling capacity of 2436.0% in 20 hrs. At pH 4.5, however, its swelling was comparable with samples cross-linked with 1 M CuCl_2_, which exhibited a lower swelling capacity ranging from 137.2% to 160.7% after 20 hrs at pH 4.5 and 433.0%–539.1% at pH 6.0 (results for pH 6.0 are clearly evident in Figures 4(b) and 4(d)). No influence on swelling capacity as a result of particle size was observed.

**Figure 5:**
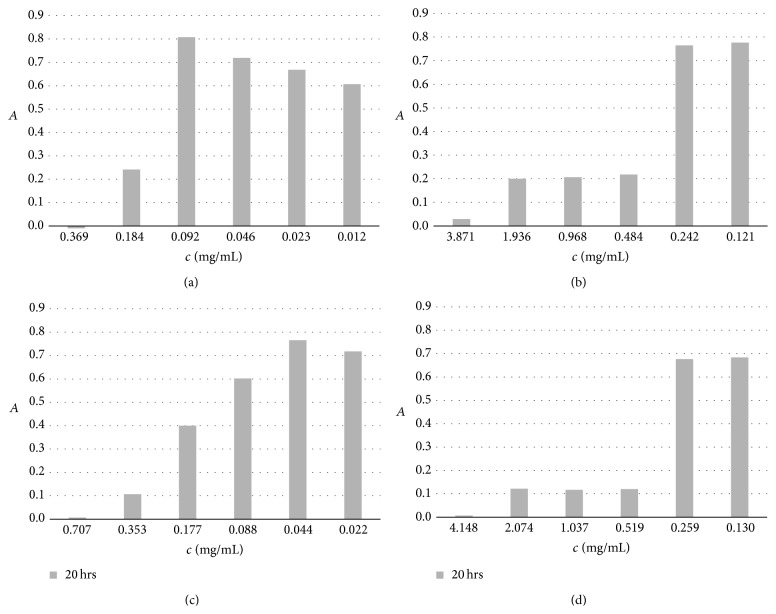
MIC results of CMC microparticles against* Escherichia coli*; (a) MP-1_0.5, (b) MP-1_1.0, (c) MP-2_0.5, and (d) MP-2_1.0.

**Figure 6:**
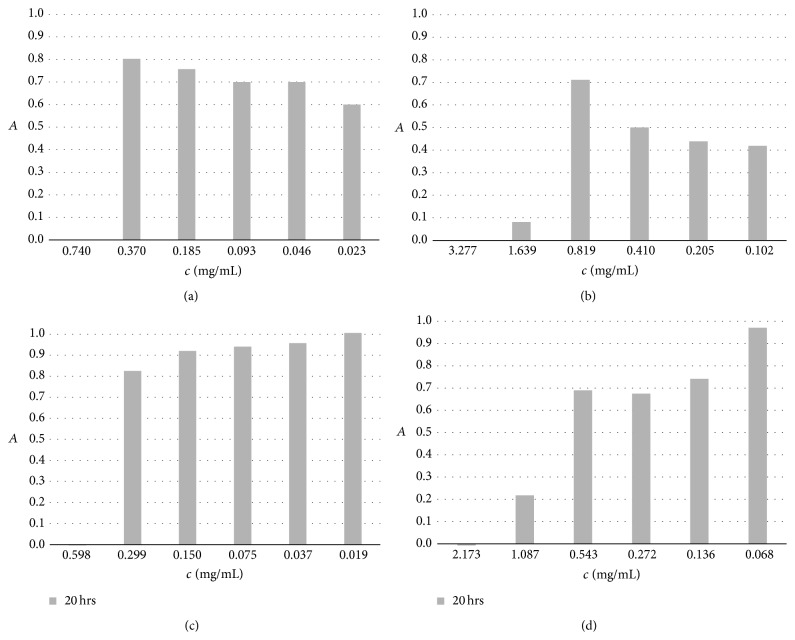
MIC results of CMC microparticles against* Candida albicans*; (a) MP-1_0.5, (b) MP-1_1.0, (c) MP-2_0.5, and (d) MP-2_1.0.

The mechanism for the antibacterial activity of Cu^+^ ions is based on their energetically easier movement across a lipid bilayer and uptake by the cell, generating reactive oxygen species, leading to lipid peroxidation and protein oxidation [35]. The excess of copper causes a decrease in the membrane integrity of a microorganism, which causes the particular cell to leak nutritional elements, like potassium and glutamate, which lead to desiccation and, ultimately, cell death [36]. The antimicrobial activity of selected CMC microparticles cross-linked by Cu^2+^ was evaluated by MIC test using Gram-negative* Escherichia coli *and* Candida albicans* yeast (see Figures 5 and 6). The test expresses the minimum concentration of antimicrobial agent that inhibits the visible growth of microorganisms [37]. The test was triplicated. For* E. coli, *MIC values after 20 hrs of incubation were 0.184, 0.484, 0.353, and 0.519 mg/mL for samples MP-1_0.5, MP-1_1.0, MP-2_0.5, and MP-2_1.0, respectively. MIC values for* C. albicans* were higher for the same samples: 0.740, 1.639, 0.598, and 1.087 mg/mL, respectively. Higher MIC values for* C. albicans* in comparison with the values for* E. coli* can be explained by the higher resistance of the yeast to the copper's antibacterial effect. Weissman et al. confirm this, reporting on the isolation of two genes involved in copper detoxification in* C. albicans*: metallothionein, CaCUP1, and a copper-transporting P-type ATPase, CaCRP1 [38]. These two genes account for* C. albicans*'s resistance to copper and consequently the vast differences in our results.

The obtained MIC values prove substantial antimicrobial activity against both microorganisms tested in this study and are in good agreement with copper MIC values against strains of* E. coli* and* C. albicans* described by different authors in other studies. Martínez Medina et al. reported MIC values for Cu(II) complexes of 0.375 mg/mL and >1.5 mg/mL for* E. coli* and* C. albicans*, respectively [39]. Copper nanoparticles prepared by Zain et al. (200 nm) showed MIC values of 0.433 mg/L for* E. coli* [40], and those prepared by Ruparelia et al. (9 nm) showed MIC values of 0.280 mg/mL for* E. coli* [41].

Despite the lower content of copper in microspheres, the samples prepared using the less-concentrated CuCl_2_ (0.5 M) solution—MP-1_0.5 and MP-2_0.5—exhibited lower MIC values and so better antibacterial activity against both tested pathogens in comparison with samples cross-linked in the more potent 1 M CuCl_2_ solution. These results confirmed the great influence of the microparticle swellability on MIC values. Both samples exhibited high swelling capacity at pH 6.0 (see Figure 3) and thus good conditions for the release of copper ions from particles (pH of LB and YNB medium was also close to 6.0). Against* E. coli*, sample MP-1_0.5 was found to be the most effective, as it probably exhibited faster copper release. This could be a result of the higher cumulation of copper ions on more rougher surface (see Figures 1(a) and 1(b)) and also smaller particle size (see Table 2) and thus larger surface area [42]. These characteristics could promote faster swelling, leading to the creation of an amorphous structure during the 20-hour interval (Figure 4(a)). Against the more resistant* C. albicans*, however, the most effective sample was MP-2_0.5, exhibiting probably more uniform copper release due to the higher CMC concentraction (2%), more uniform copper distribution in the microspheres, and larger particle size (see Table 2). This theory is also supported by the fact that the MP-2_0.5 microparticles maintained their shape-specific structure after 20 hrs of swelling (Figure 4(c)).

## 4. Conclusion

In this experimental work, CMC dispersions of two different concentrations (1% and 2%) were cross-linked by copper (II) ions (0.5, 1, 1.5, or 2.0 M CuCl_2_) to prepare microspheres with antimicrobial activity against frequently occurring vaginal pathogens* Escherichia coli* and* Candida albicans*. All tested samples exhibited stable but pH responsive behaviour in environments corresponding with natural and inflamed vaginal conditions and proved substantial antimicrobial activity against both pathogens. The most effective samples were those hardened in a less-concentrated CuCl_2_ (0.5 M) solution. Unexpectedly, a crucial parameter for microsphere antimicrobial activity was not found in the copper content but in the swelling capacity of the microparticles and in the degree of CMC surface shrinking. The sample prepared using a 1% CMC dispersion cross-linked by 0.5 M CuCl_2_ seemed to be the most suitable for potential vaginal use not only due to its antibacterial activity but also due to its gradual change to a nonspecific-shaped gel which is preferable when considering vaginal dosage forms.

## Figures and Tables

**Table 1:** Variables during preparation of microparticles samples.

Sample	CMC concentration (%)	Molarity of CuCl_2_ (mol/dm^3^)
MP-1_0.5	1	0.5
MP-1_1.0	1	1.0
MP-1_1.5	1	1.5
MP-1_2.0	1	2.0
MP-2_0.5	2	0.5
MP-2_1.0	2	1.0
MP-2_1.5	2	1.5
MP-2_2.0	2	2.0

**Table 2:** Microparticle characteristics: equivalent diameter, sphericity factor, and copper content.

Sample	ED (*μ*m)	SD (*μ*m)	SF	SD	Copper content (g/kg)	SD (g/kg)
MP-1_0.5	775.7	30.2	0.906	0.044	101.3	0.65
MP-1_1.0	888.5	58.4	0.934	0.038	143.6	2.31
MP-1_1.5	738.1	30.3	0.887	0.056	152.3	0.19
MP-1_2.0	798.6	87.9	0.855	0.059	173.0	2.15
MP-2_0.5	1016.9	22.8	0.891	0.040	143.6	0.26
MP-2_1.0	1078.0	12.4	0.886	0.071	164.7	0.40
MP-2_1.5	922.1	57.6	0.850	0.067	187.6	1.32
MP-2_2.0	933.6	38.6	0.874	0.057	200.3	1.03
